# Standardizing therapeutic parameters of acupuncture in vascular dementia rats

**DOI:** 10.1002/brb3.1781

**Published:** 2020-08-06

**Authors:** Na‐Na Yang, Si‐Ming Ma, Jing‐Wen Yang, Tian‐Ran Li, Cun‐Zhi Liu

**Affiliations:** ^1^ Acupuncture Research Center School of Acupuncture‐Moxibustion and Tuina Beijing University of Chinese Medicine Beijing China; ^2^ Beijing Institute of Traditional Chinese Medicine Beijing Hospital of Traditional Chinese Medicine affiliated to Capital Medical University Beijing China

**Keywords:** acupuncture, neuronal damage, parameters, vascular dementia

## Abstract

**Introduction:**

Despite acupuncture having been successfully used for the clinical treatment for vascular dementia in Asian countries for centuries, scientifically rigorous evidence is lacking for standardizing therapeutic parameters. To address this problem, it is necessary to examine the parameters of acupuncture using scientific methodology. The goal of this study is to investigate various therapeutic parameters, including manipulation, retention, and frequency of acupuncture, and their contribution to the efficacy of acupuncture in VD.

**Methods:**

We selected needle retention, treatment frequency, and needle rotation as the parameters. Acupuncture was performed on acupoints ST36 and GV20. Morris Water Maze was selected to assess the effect of acupuncture on cognitive function and Nissl staining indicated the hippocampal neuronal damage in VD rats.

**Results:**

Acupuncture stimulation resulted in a significant improvement in the spatial learning and memory deficit and reversed neuronal damage in the hippocampus. The approach of needle retention with 10 min, rotation for 30s every 5 min or daily treatment with acupuncture was more effective than nonretention, nonrotation, or alternative day treatment group.

**Conclusion:**

This study demonstrated that needle retention, treatment frequency, and needle manipulation are important factors in improving the learning and memory as well as reducing the neuronal damage of the hippocampus in a rat VD model. These findings strongly suggest that the appropriate therapeutic parameters could significant influence the efficiency in animal experiments.

## Abstract 2

A series of appropriate therapeutic parameters, including rotating, retaining and frequency, could strongly influence the effectiveness of acupuncture in VD. Provide a new treatment plan that manipulation with twisting‐twirling, retaining needle at least 10 min and once a day treatment after 2VO surgery 1 day or 3 days for future experiments of VD rat.

## 1 INTRODUCTION

Vascular dementia (VD) is a heterogeneous group of brain disorders associated with loss of memory and cognitive function resulting from vascular lesions. It is the second leading cause of cognitive impairment after Alzheimer's disease (AD) in the elderly (Du et al., 2013). In developing countries, VD accounts for approximately 30% of the age‐adjusted dementia prevalence and in Western countries (Kalaria et al., 2008), the rate of VD is approximately1%‐4% in people at the age of 65 (McVeigh & Passmore, 2006). The WHO estimates that 50 million people have dementia, a number that is anticipated to triple by 2050. As people live longer, the burden of cognitive impairment caused by VD will become increasingly important (Gorelick et al., 2011).

The importance of VD has gradually increased over the past few decades, however, effective treatment remains elusive. The traditional Chinese medicine treatment of acupuncture has a history of >2000 years and is becoming an increasingly important therapy for various diseases worldwide. In acupuncture, a thin needle is placed into an acupoint and manually manipulated in different ways including thrusting, lifting, twisting, twirling, or combination of these motions. So far, various clinical trials (Zhou, Peng, Xu, Li, & Liu, 2015) and animal experiments (Du et al., 2018; Li et al., 2015, 2016; Xiao et al., 2018) indicated that acupuncture was effective to improve the cognitive function in VD. We have demonstrated that acupuncture improved learning and memory impairment through anti‐oxidative and anti‐apoptotic mechanisms in VD rats (Zhu et al., 2018), and this therapeutic effect is associated with the selection and combination of acupoints. We demonstrated that acupuncture at “ST36 + GV20” was more effective than “ST36 + SP10” or “GV20 + SP10” in VD rats (Ye et al., 2017). Most research has focused on acupoint specificity, less on exploring the effect of acupuncture manipulation (K. Wang et al., 2020).

It is noticeable that needle retention, treatment frequency, and needle manipulation are the core method of acupuncture according to traditional Chinese medicine theories, which are both covered in the studies on the optimal stimulation of acupuncture (Dimitrova, Murchison, & Oken, 2017; Yu et al., 2014). Acupuncture with a 10 min retention of the needle has been commonly used and had therapeutic effects on cognitive function in VD rats (Ye et al., 2017). However, many studies indicated that acupuncture without needle retention had similar beneficial effects (Yang et al., 2014). Therefore, in a VD experiment, needle retention is controversial. Manual manipulation is another key factor that influences the therapeutic effects of acupuncture and includes simple reinforcing and reducing manipulation, such as rotation and thrusting. However, the rotation is the most commonly used method in clinical practice (Lu et al., 2017) and was more suitable for using in GV20 acupoints which is weak tissue of the body. In addition, almost all of the studies that used manual acupuncture adopted 30s as their manipulation duration (Wang, Liu, Yu, Jiang, & Han, 2009; Yu, Liu, Zhang, & Han, 2005) and two times manipulation were usually adopted during clinical acupuncture. Based on these results, acupuncture with 30s manipulation every 5 min is suitable for studying the effects of rotation in VD rats.

This study refined the parameters of needle retention, treatment frequency, and needle manipulation as individual therapeutic parameters. This study aimed to determine whether individual stimulation parameters affect the therapeutic effect of acupuncture to enable standardizing therapeutic parameters of acupuncture in VD rats. Our results may highlight the importance of therapeutic parameters in acupuncture and strengthen the effectiveness of acupuncture in clinical application.

## 2 MATERIALS AND METHODS

### 2.1 Animals

Adult male Wistar rats (270‐300 g) were purchased from the Vital River Laboratory Animal Technology Co. Ltd. (Beijing, China). Rats were housed under a controlled temperature at 22 ± 2°C and were kept on 12 hr dark/light artificial cycle with food and water ad libitum. A three days acclimaization period was done before the start of the surgery. The animal studies were approved by the Guidelines for Animal Experimentation of the Capital Medical University (no.2015040801), and all procedures were performed according to the guidelines of the National Institutes of Health Guide for the care and Use of Laboratory Animals.

### 2.2 Surgery

A standardized permanent bilateral common carotid artery occlusion (2VO) procedure was done as described elsewhere to induce the VD model (Zhu et al., 2018). Briefly, animals were fasted for 24h prior to 2VO surgery. Rats were anesthetized by sodium pentobarbital (40 mg/kg, i.p.), and then a midline incision was made to expose the common carotid arteries. The common carotid arteries were separated from the vagus nerve in all groups and then double ligated with silk sutures. Rats in the sham group had the same operation without occlusion. After surgery, the skin incisions were closed by sutures and the rats were returned to their cages with free access to food and water.

### 2.3 Sample size

We determined the sample size via our previous animal experiments of acupuncture treatment for VD rats. The time spending in the target quadrant was primary outcome in the VD group and acupuncture group. Our previous study evaluated the effects of acupuncture for 2 weeks in the VD rats (Wang et al., 2015). The change of the time spending in the target quadrant after treatment was 20.72 ± 1.67 in the VD group and 24.05 ± 1.53 in the acupuncture group. The required number of participants per group was 4, considering a 2‐tailed test with a 1:1 allocation ration, a test power of 80% (1‐β) and a significance level of 5% (α). According to the result of sample size calculation, 6 rats for MWM and 6 rats for Nissl staining are feasible in this experiment.

### 2.4 Experiment design

#### 2.4.1 Experiment Ⅰ

To observe the effect of needle retention on cognitive function and hippocampal neuronal damage in VD rats, 48 rats were randomized into four groups (*n* = 12 each group): A sham group, 2VO group (without treatment), needle retention group (2VO + acupuncture with 10 min of needle retention), and a nonretention group (2VO + acupuncture without needle retention). The sham and 2VO group did not receive treatment. The other groups received acupuncture treatment for 14 days, with a rest on the seventh day, for a total 12 treatments.

After treatment, 6 rats in each group were subjected to the Morris Water maze (MWM) and others were subjected to Nissl staining after sacrifice.

### 2.5 Experiment Ⅱ

To further assess the role of different stimulating frequencies of acupuncture on improving cognitive impairment, VD rats were randomly divided into two groups (*n* = 12 each group): 1DT (daily treatment) and 2DT (alternative day treatment). In the 1DT group, acupuncture treatment was similar to the needle retention group in experiment I, for a total 12 treatments over two weeks. In the 2DT group, acupuncture was performed every two days during the two weeks of treatment, with a rest on the seventh day, for a total 6 treatments. The needles were retained for 10 min in two groups.

### 2.6 Experiment Ⅲ

In order to investigate whether needle manipulation influenced on the outcomes of VD rats, the rats were divided into two groups (*n* = 12 each group): AM (acupuncture with 30s manipulation every 5 min, rotation angle <90°and rotation frequency >120 times/min) and ANM (acupuncture without manipulation). The two groups received 10 min’ needle retention and two weeks’ daily treatment, for a total 12 treatments.

### 2.7 Acupuncture treatment

Three days after 2VO surgery, rats in the acupuncture groups received acupuncture treatment for 2 weeks at the acupoints GV20 and bilateral ST36. GV20 is located above the apex auriculate, on the midline of the head, while ST36 is located at the proximal one‐fifth point on the line from the depression lateral to the patella ligament to the anterior side of ankle. Acupuncture needles (Hwato and china, 0.3 × 40 mm) were used and inserted into 5 mm at acupoints. The acupuncture methods in detail are shown in Table 1. The rats in sham group and 2VO group were performed the same time and level catching‐grasping stimulus as the acupuncture group.

**Table 1:** Groups and acupuncture operation

	Groups	Acupuncture Operation
Ⅰ	Retaining	Every day + 10 min retention
	Nonretaining	Every day + nonretention
Ⅱ	1DT	Every day + 10 min retention
	2DT	Every two days + 10 min retention
Ⅲ	AM	Every day + 30s manipulation every five minutes + 10 min retention
	ANM	Every day + nonmanipulation+10min retention

### 2.8 Morris water maze

The first day after acupuncture treatment, the MWM was performed to detect changes in spatial learning and memory performance in the different groups of rats as previous described (Du et al., 2018). Animals were placed in a black circular and 160 cm diameter pool (depth 40 cm) which was filled with water at 23 ± 1°C. The 10 cm diameter escape platform was submerged and used for testing. After a 5 days training period, the rats were released into the water facing the wall at 3 different locations and allowed to find the hidden platform below the water surface within 90s. If a rat did not find the platform in time, it was guided to the platform and allowed to stay there for 10s. One day after the last training session, rats were placed into maze for 60s in the absence of the platform. The time that each rat spent in the target quadrant and the number of times it crossed the different quadrant platforms were recorded. All data are collected with the Top Scanlite (USA) tracking program. After completion of the MWM test, rats were sacrificed by cervical dislocation after deep anesthesia with sodium pentobarbital.

### 2.9 Nissl staining

The first day after acupuncture treatment, the rats were sacrificed and the left ventricle exposed and perfused with 0.9% saline followed by 4% paraformaldehyde (PFA, pH7.4). Excised brains and the 10‐μm‐thick coronal sections of the hippocampus were cut. The sections were incubated with 1% cresyl violet (Sigma‐Aldrich, St.Louis, MO,USA) solution at 50℃ and then dehydrated in graded alcohol solutions (70%, 80%, 90%, and 100%), followed by immersion in xylenes, mounted in neutral balsam, and covered with a coverslip. Nissl‐positive cells in the pyramidal layer of medical CA1 were observed by a visible microscope for neuronal loss.

### 2.10 Data analysis and statistics

Data were reported as the mean ± standard error of mean (*SEM*). The data of the MWM experiments, including escape latency, swimming speed, and direction, were analyzed by a two‐way repeated‐measures analysis of variance (ANOVA) (group/day), followed by the least‐significant difference (LSD) test for multiple comparisons among various groups. Difference in the other data was analyzed using a one‐way ANOVA, followed by the LSD test for intergroup comparisons. A probability value (*P*) of less than 0.05 was considered significant. GraphPad Prism V.5.01 software was used to perform statistical analysis.

## 3 RESULTS

### 3.1 Acupuncture with needle retention had a significant therapeutic effect of cognitive impairment in VD rats

**Figure 1:**
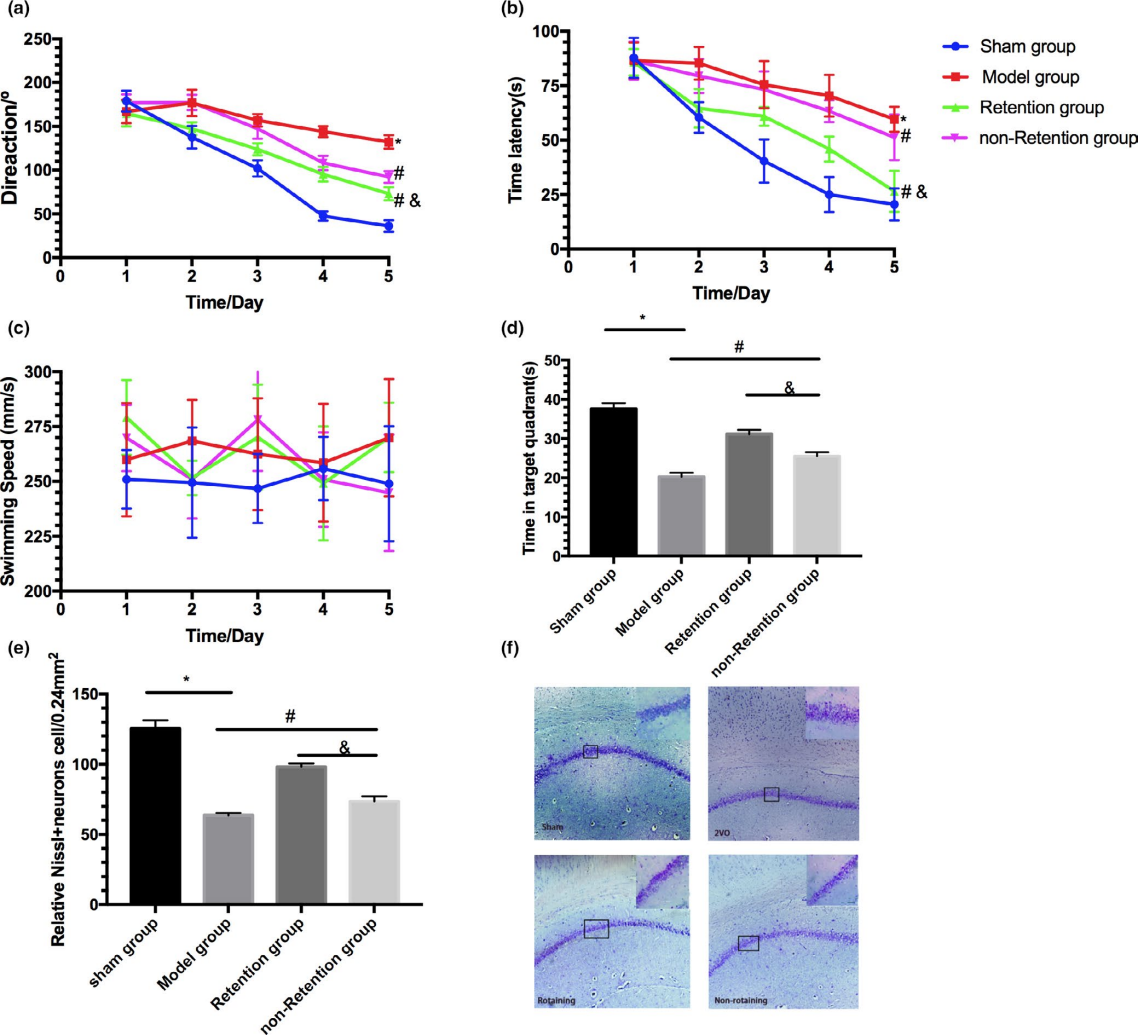
The function of retention in acupuncture ameliorating 2VO‐induced learning and memory deficits. (a) Direction. (b) Time latency. (c) Swimming speed. (d) The time spent in target quadrant on the sixth day. (e and f) Nissl staining. Data are presented as mean X ± *SEM* (*n* = 6 in each group). *p < .001 versus Sham group; #*p* < .05 versus model group; &*p* < .05 versus nonretention group

To assess whether needle retention improves learning and memory impairment in VD rats, direction, and escape latency, the time spending in the target quadrant and swimming speed were measured in a MWM experiment. It was significantly different over time (*F* = 13.62, *p* < .05; *F* = 53.85, *p* < .05) and for treatment (*F* = 23.67, *p* < .05; *F* = 34.96, *p* < .05); the interaction of time and treatment was not significant (*F* = 78.52, *p* > .05; *F* = 85.86, *p* > .05) in the direction and escape latency. There was a significant increase in direction and escape latency in the 2VO group compared with the sham group (*p* < .001; Figure 1a‐b), which was reversed after acupuncture (*p* < .05) in the needle retention group and nonretention group. The time that the rats spent in the target quadrant were significantly decreased in the 2VO group compared with the sham group (*p* < .01; Figure 1d). Acupuncture treatments markedly increased the time in the target quadrant (*p* < .01) in both groups undergoing that intervention. No significant differences were found in swimming speed between these four groups (*p* > .05; Figure 1c).

Nissl staining demonstrated that hippocampal neuronal damage and death (including neuronal lose, shrivelling, and disarranging) in the 2VO group were more serious than that in the sham group (*p* < .001; Figure 1e‐f). Acupuncture significantly reversed these neuronal damaging in the hippocampal CA1 region, and this attenuation was more effective in the needle retention group than in nonretention group (*p* < .05).

### 3.2 The effectiveness of acupuncture was related to the frequency in VD rats

**Figure 2:**
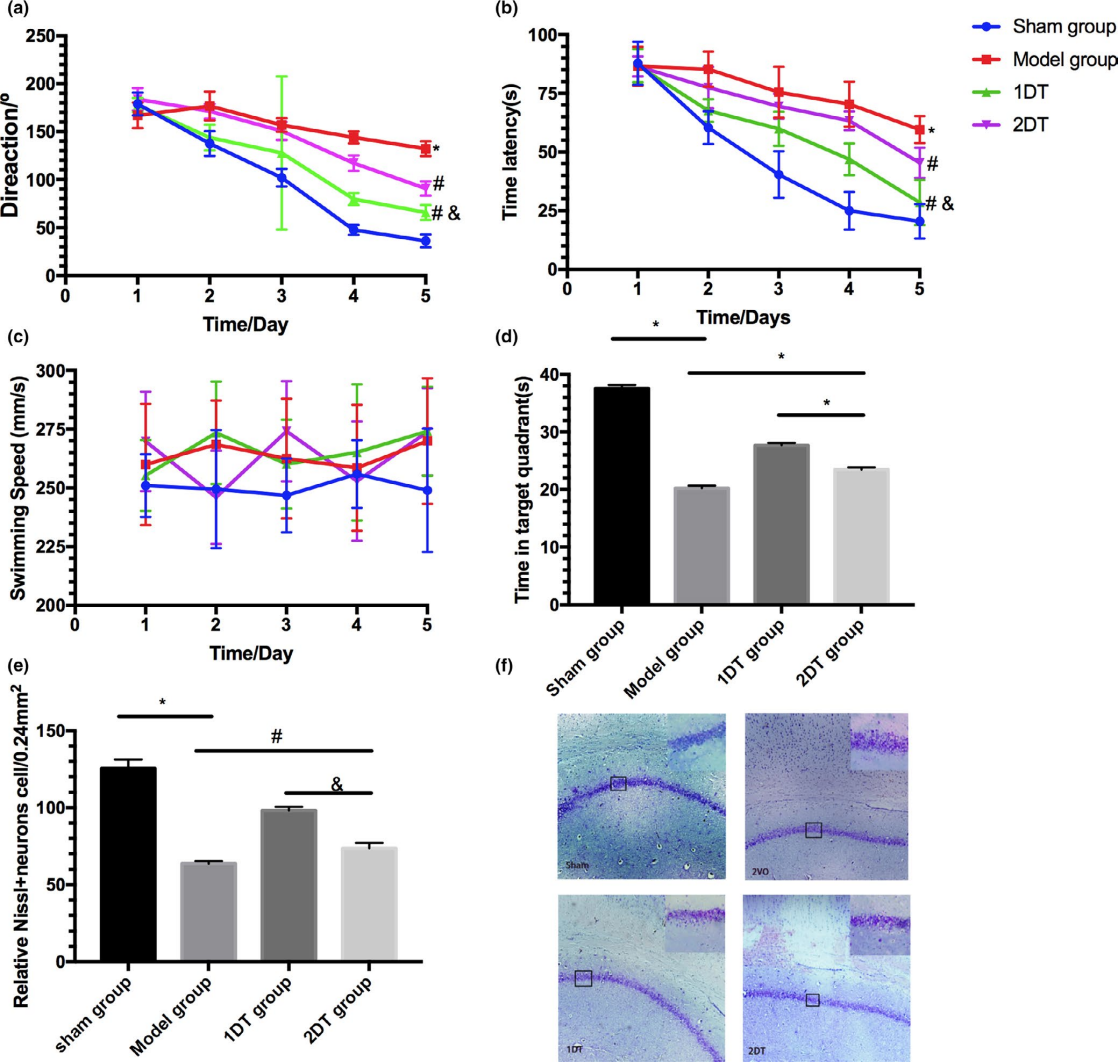
The function of frequency in acupuncture ameliorating 2VO‐induced learning and memory deficits. (a) Direction. (b) Time latency. (c) Swimming speed. (d) The time spent in target quadrant on the sixth day. (E and F) Nissl staining. Data are presented as mean X ± *SEM* (*n* = 6 in each group). *p < .001 versus Sham group; #*p* < .05 versus model group; &*p* < .05 versus 2DT group

We next evaluated the effect of different acupuncture frequencies on cognitive impairment in VD rats. The MWM results demonstrated that there was a significant difference over time (*F* = 14.55, *p* < .05; *F* = 58.43, *p* < .05), treatment (*F* = 25.93, *p* < .05; *F* = 38.45, *p* < .05); the interaction of time and treatment was not significant (*F* = 80.03, *p* > .05; *F* = 88.47, *p* > .05) regarding direction and escape latency. The time in the 2VO group was longer in direction and escape latency and was shorter in the target quadrant compared with the sham group (*p* < .001; Figure 2a‐d), which were reversed by acupuncture (*p* < .05). Notably, the daily treatment group showed a longer time in the target quadrant and a shorter time in the direction and escape latency compared with every other day treatment (*p* < .05). There was no significant difference in swimming speed between four groups (*p* > .05, Figure 2c).

The pathological results showed that the number of neurons in the CA1 region of the hippocampus was decreased significantly after 2VO compared with the sham group (*p* < .001; Figure 2e‐f). Acupuncture alleviated neuronal damage in both the 1DT group (*p* < .05) and 2DT group (*p* < .05), and the 1DT group showed less cell death in the hippocampus compared with the 2DT group (*p* < .01).

### 3.3 Acupuncture with manipulation was more effective in the learning and memory impairment in VD rats

**Figure 3:**
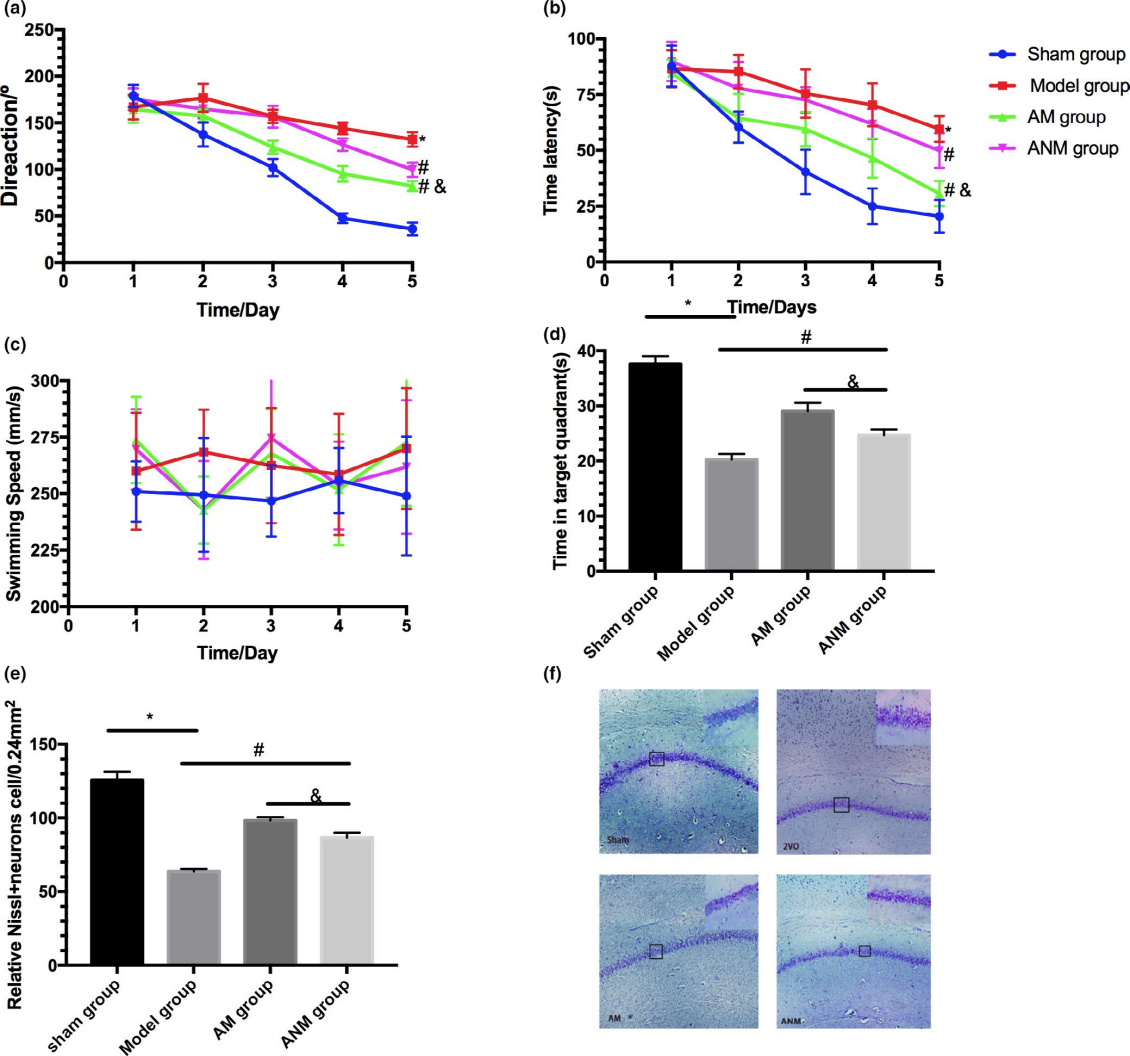
The role of manipulation in acupuncture ameliorating 2VO‐induced learning and memory deficits. (a) Direction. (b) Time latency. (c) Swimming speed. (d) The time spent in target quadrant on the sixth day. (e and f) Nissl staining. Data are presented as mean ± *SEM* (*n* = 6 in each group). *p < .001 versus Sham group; #*p* < .05 versus model group; &*p* < .05 versus ANM group

We investigated whether the manipulation might influence the effectiveness of acupuncture in VD rats. Time (*F* = 14.89, *p* < .05; *F* = 55.47, *p* < .05) and treatment (*F* = 21.56, *p* < .05, *F* = 39.73, *p* < .05) were significantly different; there was no impact of time and treatment on the direction and escape latency (*F* = 77.45, *p* > .05; *F* = 83.25, *p* > .05). Treatment with acupuncture significantly decreased the time spent in the direction and escape latency and increased the time in the target quadrant compared with 2VO rats (*p* < .01; Figure 3a‐d). Acupuncture with manipulation showed a better therapeutic effect on MWM compared with acupuncture without manipulation (*p* < .05). No difference was observed in swimming speed (*p* > .05, Figure 3a‐d). Consistently, Acupuncture alleviated neuronal damage compared with 2VO group (*p* < .01), and the acupuncture with manipulation group showed less cell death in the hippocampus compared to the acupuncture without manipulation group (*p* < .05, Figure 3e‐f).

## 4 DISCUSSION

The current experiments demonstrated that acupuncture improved cognitive function and attenuated the neuronal damage of hippocampus in VD rats. These treatment effects of acupuncture were influenced by the parameters of rotation, needle retention, and manipulation. These results highlight the importance of individual therapeutic parameters in the clinical application of acupuncture.

A more significant therapeutic effect of acupuncture was observed in the needle retention group compared with the nonretention group, which indicated that the needle retention was more effective in improving cognitive impairment and suppressing the neuronal cell damage. Needle retention is one of the most important therapeutic parameters of acupuncture. Clinical research has been found that 60 min of needle retention was more effective than 20 min or 40 min needle retention in stroke patients (He et al., 2006). Needle retention for 10 min, therefore, may not be the optimal retention time in VD rats and more parameter options should be studies in the future, such as 5 min or 15 min needle retention. The purposed of needle retention is to wait and regulate ˝qi,˝ a vital force flowing throughout the body, and then strengthen the therapeutic effect, dispel the pathogenic factors. However, that does not mean that the longer retention, could produce a better effectiveness. In the future, it will be necessary to discover the optimal needle retention time of acupuncture in VD.

Acupuncture frequency, also known as the acupuncture interval, is the length of time between two acupuncture treatments. The frequency of acupuncture is one of the main factors which affects the efficacy of acupuncture. According to our results, daily treatment of acupuncture was more effective than alternative day treatment which is similar to the findings of Qian XP, who suggested that more frequent was superior to less frequent treatment for the recovery stage of cerebral infraction (Qian, Xu, Song, & Zhao, 2009). Like a drug that acts only when reach a certain plasma concentration is achieved, acupuncture may be effective only when a certain “dosage” is reached. Therefore, once a day or two times a day may be a good choice to improve the efficacy of acupuncture in VD.

Additionally, the manipulation of the acupuncture may affect the efficacy of acupuncture. In our study, manipulation with twisting twirling showed a better effect on cognitive impairment compared with nonmanipulation. A similar observation has been documented by Huang (Huang et al., 2013), who found that needle with twisting manipulation activated more widespread brain areas compared with tactile stimulation. Another study demonstrated that acupuncture with manipulation was superior to drug treatment in regulating blood vessels and improving depression and anxiety in stroke patients (Yu et al., 2014). In these studies, manipulation strengthened the effect of acupuncture in changing skin temperature, increasing the blood circulation, exciting the sympathetic nerves, and promoting metabolism of the tissues.

The therapeutic effect of acupuncture is closely related to the amount of stimulation which is often difficult to be determined in clinical studies. The reason is that there are many parameters affecting the stimulation amount, including manipulation selection, treatment time, needle velocity, and force (Lyu, Gao, Yang, Wen, & Tang, 2019). So, more parameters should be further investigated, except for needle retention, stimulation frequency, and rotation.

In conclusion, the therapeutic effects of acupuncture in VD rats were influenced by different acupuncture parameters, including needle retention, treatment frequency, and needle manipulation. In light of the results of this study, a new and comprehensive treatment plan is proposed, including manipulation with twisting twirling, needle retention for at least 10 min and once a day treatment after 2VO surgery for future experiments of VD rats.

## CONFLICT OF INTEREST

The authors declare no conflict of interest.

## AUTHORS' CONTRIBUTIONS

Jing‐Wen Yang and Cun‐Zhi Liu were involved in the study design. Tian‐Ran Li performed the experiments. Na‐Na Yang contributed to the analysis and interpretation of data. Na‐Na Yang and Si‐Ming Ma wrote the manuscript. All the authors revised critically and approved the final version of the manuscript and agree to be accountable for all aspects of the work in ensuring that questions related to the accuracy or integrity of any part of the work are appropriately investigated and resolved. All persons designated as authors qualify for authorship, and all those who qualify for authorship are listed.

### Peer Review

The peer review history for this article is available at https://publons.com/publon/10.1002/brb3.1781.

## Data Availability

The data that support the findings of this study are available from the corresponding author upon reasonable request.
